# Congenital portosystemic venous shunt associated with 22q11.2 deletion syndrome: a case report

**DOI:** 10.1186/s12887-022-03447-3

**Published:** 2022-06-29

**Authors:** Toshinobu Ifuku, Sayo Suzuki, Yusaku Nagatomo, Ryohei Yokoyama, Yoshiko Yamamura, Keigo Nakatani

**Affiliations:** 1 Department of Pediatrics, Miyazaki Prefectural Miyazaki Hospital, 5-30, Kita Takamatsu-cho, Miyazaki City, Miyazaki, 880-8510 Japan; 2 Department of Cardiology, Fukuoka Children’s Hospital, Fukuoka, Japan; 3 Department of Pediatrics, Kyushu University Hospital, Fukuoka, Japan

**Keywords:** Portosystemic shunt, 22q11.2 deletion syndrome, Congenital heart disease, Case report

## Abstract

**Background:**

22q11.2 deletion syndrome (22qDS) is the most common chromosomal microdeletion syndrome and is associated with a high rate of congenital heart disease (CHD) and neurodevelopmental abnormalities. Congenital portosystemic venous shunts (CPSS) are rare developmental abnormalities of the portal venous system. The clinical manifestations of CPSS are varied, and some patients have CHD or genetic chromosomal abnormalities, but their relationship remains unknown. We report the first case of CPSS associated with 22qDS.

**Case presentation:**

A newborn boy referred to our institution was diagnosed with 22qDS due to characteristic facial features and complications of tetralogy of Fallot. A subsequent newborn screening test indicated hypergalactosemia and high blood levels of ammonia and bile acids. Upon closer examination, these abnormalities were found to be caused by the CPSS. Abdominal contrast-enhanced computed tomography and angiography confirmed that abnormal blood vessels ascended from the splenic vein and short-circuited to the left renal vein. Intracardiac repair for CHD was performed at 1 year of age, followed by transcatheter occlusion of the CPSS using a multilayer device (vascular plug) and detachable coil at 2 years of age. After treatment, the abnormal blood parameters promptly normalized.

**Conclusions:**

As the blood flow of CPSS bypasses the liver, the levels of galactose, bile acids, and ammonia in the systemic veins can increase. Some patients with CPSS have CHD, and these toxic substances may cause liver and lung lesions as well as portosystemic encephalopathy (PSE). Several genetic chromosomal abnormalities, including 22qDS, and CPSS have similar symptoms, and neurodevelopmental abnormalities, particularly those caused by PSE, may be difficult to diagnose. Blood tests, such as newborn screening, and abdominal imaging are useful in the early diagnosis of CPSS.

## Background

22q11.2 deletion syndrome (22qDS) is the most common chromosomal microdeletion syndrome and occurs in 1 in 3000–6000 individuals [1–3]. The clinical phenotype is diverse, with complications ranging from mild to severe. In the neonatal period, congenital heart disease (CHD) affects prognosis. From infancy onward, neurological symptoms, such as developmental delays and psychiatric disorders, are often problematic [3–5].

Congenital portosystemic venous shunts (CPSS), in which portal blood flows into the systemic venous system through abnormal shunt vessels, are rare developmental abnormalities of the portal venous system during fetal life. The overall prevalence of CPSS is reported to be 1 in 30,000, and the rate of shunt vessels remaining open after infancy is 1 in 50,000 [6]. CPSS are classified into intrahepatic and extrahepatic types and are often associated with CHD [6–9]. The concentrations of galactose, bile acids, ammonia, and manganese in the systemic veins increase when shunt blood bypasses the liver. Hence, some patients with CPSS develop portosystemic encephalopathy (PSE) and a variety of neurological symptoms [9]. Symptoms of CPSS are influenced by abnormal vessel size and short-circuit blood flow and may present with a variety of clinical symptoms or even be asymptomatic. The age and trigger for being diagnosed with CPSS varies from case to case.

Here, we report a case of 22qDS diagnosed as CPSS secondary to hypergalactosemia in the neonatal period. To the best of our knowledge, complications of these two diseases have never been reported.

## Case presentation

A late preterm (35 weeks of gestation) baby boy weighing 2064 g was delivered by cesarean section. He had peculiar facial features and CHD, for which he was referred to our center for further management. His mother had been taking levothyroxine for Hashimoto’s thyroiditis, and her thyroid function was stable. No family history of CHD or genetic abnormalities was noted.

On admission, physical examination revealed dysmorphic features of hypertelorism, a broad nose bridge, low set ears, a long philtrum, and a thin upper lip. A 2/6 systolic ejectional murmur was best heard at the lower left-sternal border. Echocardiography demonstrated tetralogy of Fallot (ToF) and a right-sided aortic arch. Ultrasonography of the head and abdomen revealed no obvious abnormalities. Based on these features, 22qDS was strongly suspected and fluorescent in situ hybridization analysis performed on peripheral blood cells showed the presence of a 3.0 Mb 22q11 deletion.

Subsequently, a routine newborn screening test on day 4 of life revealed hypergalactosemia (3.7–6.8 mg/dl; normal: < 3.0 mg/dl). In addition, the levels of blood bile acids, ammonia, and manganese were mildly elevated. Both galactose-1-phosphate, assessed by an enzymatic assay, and galactose-1-phosphate uridyltransferase (GALT) activity, assessed by the Beutler spot assay, were within the normal range. Citrin deficiency was ruled out by blood and urine amino acid analyses.

**Fig. 1:**
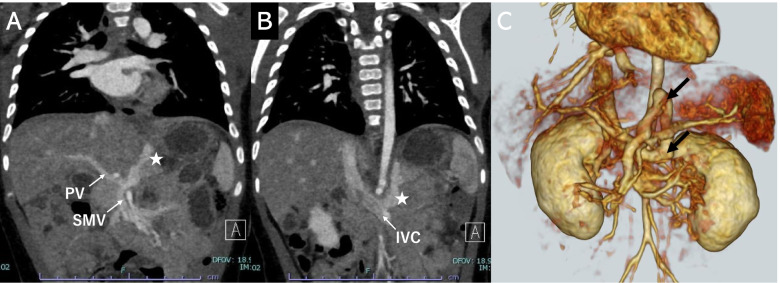
Abdominal contrast-enhanced computed tomography (CT). **A** The CPSS branches from the splenic vein and rises once cephalad. **B** The CPSS arches and descends at a level close to the stomach and flows into the left renal vein. **C** Detailed morphology of the CPSS (arrow) on three-dimensional CT. CPSS, congenital portosystemic venous shunt; PV, portal vein; SMV, superior mesenteric vein; IVC, inferior vena cava

**Fig. 2:**
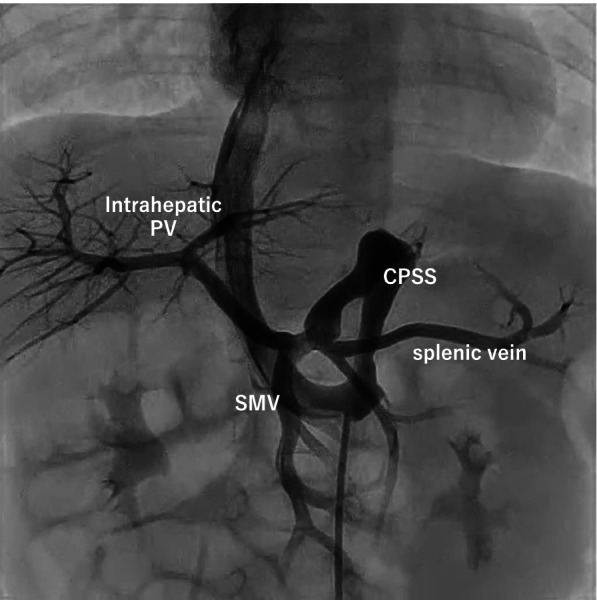
Abdominal angiography showing good development of the intrahepatic portal vein (PV). The pigtail catheter reaches the CPSS from the inferior vena cava through the left renal vein

Abdominal contrast-enhanced computed tomography (CT) revealed a CPSS (Fig. 1). Abnormal blood vessels branching from the splenic vein were observed, which initially ascended and then descended to join the left renal vein. A left-sided inferior vena cava (IVC) was also evident. Although the blood flow of the superior mesenteric vein was partially short-circuited to this abnormal vessel, angiography revealed relatively good development of the intrahepatic portal vein (Fig. 2). No neoplastic or vascular lesions of the liver or biliary atresia were observed. Brain magnetic resonance imaging (MRI) showed no abnormal signals in the brain parenchyma or any vascular lesions.

Hypergalactosemia and other blood test abnormalities were diagnosed as caused by extrahepatic CPSS, which is a short circuit to the body veins in front of the portal vein. Galactose-free milk and oral lactulose syrup were started to reduce elevated galactose and ammonia levels in the blood. During hospitalization, the baby’s nutritional status was good, and his serum electrolyte and calcium levels were within the normal range.

The patient was discharged at 1 month of age and transitioned to outpatient follow-up. Blood galactose levels decreased and normalized during the first few months of life. Galactose-free milk was discontinued after 6 months of age. During infancy, the blood ammonia level varied between 40 and 119 μmol/l (normal: 38–80 μmol/l), and there were no findings of hepatic encephalopathy, portal hypertension, or pulmonary hypertension. His SpO_2_ was in the 90% range, and he did not have any cyanotic spells. We decided to proceed with treatment for ToF. After weight gain was achieved, intracardiac repair for ToF was performed at 1 year of age. Postoperatively, cyanosis disappeared, but mild pulmonary valve regurgitation and branch pulmonary artery stenosis remained.

**Fig. 3:**
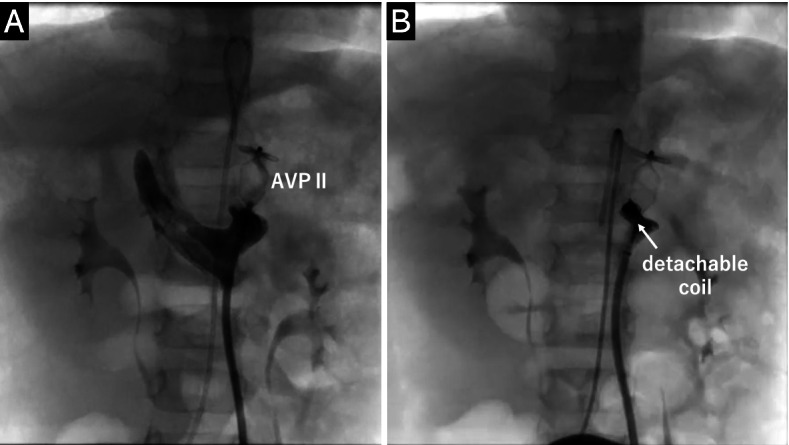
Transcatheter occlusion of the CPSS at 2 years of age. **A** Angiography showing a residual leak after implanting a 12-mm AMPLATZER Vascular Plug II (AVP II) in the CPSS. **B** The detachable coil additionally filled the disk part of AVP II, and the leak disappeared. CPSS, congenital portosystemic venous shunt

At the age of 2 years, he underwent transcatheter occlusion of the CPSS. Left femoral vein access was achieved, as it is the straightest route through the left IVC, left renal vein, and abnormal vessels. A 12-mm AMPLATZER Vascular Plug II (AVP II; AGA Medical Corporation, Plymouth, MN, USA) was advanced on a 0.035-J guidewire through a 6-F Destination® guiding sheath (Terumo Corporation, Tokyo, Japan). The plug size was based on the diameter of the vein in the target segment obtained by CT and angiography **(**Fig. 3A**)**. As a small leak remained, an additional 5 × 200-mm Target XL® detachable coil (Stryker Neurovascular, Fremont, CA) was inserted between the discs of the AVP II to completely occlude abnormal vessel flow **(**Fig. 3B**)**.

The procedure was uneventful, and his blood ammonia, bile acid, and manganese levels normalized within two weeks. At the 1- and 3-month follow-ups, the patient was asymptomatic, and abdominal radiography and ultrasound showed no evidence of device displacement or residual leak. His motor development was mildly delayed since infancy, and he was unable to speak meaningful words at the age of two. He continued to receive developmental therapy at a local facility.

## Discussion and conclusions

We reported the first case of an association between 22qDS and CPSS. In this case, the diagnosis of 22qDS was relatively easy because of the combination of characteristic facial features and CHD. However, CPSS was incidentally diagnosed due to hypergalactosaemia. The prevalence of CHD in 22qDS is approximately 75–80%, with conotruncal defects like ToF accounting for nearly half of the cases [2, 3]. In infants and toddlers, gross and fine motor difficulties, expressive language delays, and speech problems become prominent [2, 10]. As the child grows, anxiety disorders, autism spectrum disorders, and schizophrenia may become apparent [11], requiring lifelong observation and individually tailored management.

CPSS are thought to be caused by remnants or excessive retraction of the left and right vitelline veins during fetal life [6, 7]. Persistent patent ductus venosus is another type of CPSS. The morphology of CPSS is diverse and is classified not only by anatomical classification but also by histology, clinical presentation, and the presence or absence of an intrahepatic portal vein, which guides subsequent treatment [6, 7, 9]. CHDs, such as ToF, atrial septal defect, ventricular septal defect, and patent ductus arteriosus, are associated with 17–23% of CPSS [12–14]. Although the link between the two diseases is unclear, some believe that hemodynamic abnormalities during fetal life caused by CPSS are involved in the development of CHD [6].

Galactose, ammonia, and bile acids are substances that undergo pronounced first-pass metabolism in the liver, and their concentration in peripheral blood increases when there is blood flow that bypasses the liver and enters the systemic circulation, such as in CPSS. Newborn screening often leads to the diagnosis of CPSS [15–17]. In Japan and other areas, newborns are screened for inherited metabolic diseases, including hypergalactosemia. Hypergalactosemia is not inevitable in CPSS and is thought to be dependent on blood flow in the shunt [16, 18]. Increased galactothiol has been reported to increase the risk of cataract [16], but whether it contributes to liver damage is unclear. Transient hypergalactosemia of unknown etiology also exists but should be differentiated from biliary atresia, citrine deficiency, Uridine diphosphate-galactose 4-epimerase deficiency, and GALT deficiency. Blood ammonia, total bile acids, alpha-fetoprotein, enzymatic analysis, and various imaging studies are useful in advancing the differential diagnosis [17].

Neurocognitive and behavioral disturbances have been reported in 17–30% of children with CPSS [6, 19]. These symptoms, called PSE, are considered to be caused by ammonia and other toxic substances that are not metabolized by the liver and circulate throughout the body. PSE is more likely to occur with age and shunt flow [19, 20]. PSE symptoms typically include lethargy and confusion after meals, abnormal behavior with irritability and disorientation, slow waves on electroencephalograms, and extrapyramidal symptoms. Brain MRI may show high signals in the bilateral globus pallidus and anterior pituitary on T1-weighted images, which are thought to be caused by manganese deposition [6, 21]. However, non-specific symptoms, such as speech delay, mental retardation, seizures, behavioral problems, and attention deficit hyperactivity disorder, may also be present [21]. Our patient had speech delay and mental retardation since infancy, but we could not distinguish whether these symptoms were due to 22qDS or CPSS.

While some intrahepatic CPSS may close spontaneously, extrahepatic CPSS and ductus venosus are unlikely to close [6, 22]. Patients with CPSS with high shunt flow and portal hypoplasia are at risk of developing PSE, hepatopulmonary syndrome, portal hypertension, and hepatic malignancy [6–8]. There is no standard therapeutic approach for CPSS. Even in the absence of obvious symptoms, early intervention can prevent serious hepatic and pulmonary complications and neurodevelopmental delays and maintain intellectual and psychosocial function [23]. Some reports recommend early shunt closure in cases of serious complications or persistent intrahepatic CPSS at 2 years of age [7–9]. Several genetic chromosomal abnormalities (e.g., trisomy 21, Turner syndrome, Noonan syndrome) have been reported to be associated with CPSS [6–9, 24]. However, their exact prevalence and genetic relationship are still unknown. CPSS and PSE may be difficult to diagnose in congenital diseases like 22qDS that have a high incidence of CHD and abnormal neurological signs. We hope that further findings on the association between CPSS and genetic chromosome abnormalities will be accumulated in the future.

In summary, CPSS is a rare but diverse disease with a wide variety of forms and clinical manifestations. CHD, and neurologic symptoms derived from PSE are complications that can be common in other diseases and can mask the presence of CPSS. Patients with unexplained neurological symptoms or developmental delays should be considered for CPSS screening with imaging studies involving the abdominal viscera and blood vessels along with investigation of blood levels of ammonia, galactose, and bile acids.

## Data Availability

Data sharing is not applicable to this article as no datasets were generated or analyzed during the current study. Data are available from the corresponding author upon request.

## Abbreviations

22qDS: 22q11.2 deletion syndrome
CHD: congenital heart disease
CPSS: congenital portosystemic venous shunt
CT: computed tomography
IVC: inferior vena cava
MRI: magnetic resonance imaging
PSE: portosystemic encephalopathy
ToF: tetralogy of Fallot

## References

[1] Kobrynski LJ, Sullivan KE (2007). Velocardiofacial syndrome, DiGeorge syndrome: the chromosome 22q11.2 deletion syndromes. Lancet..

[2] McDonald-McGinn DM, Sullivan KE (2011). Chromosome 22q11.2 deletion syndrome (DiGeorge syndrome/velocardiofacial syndrome). Med (Baltim).

[3] Cirillo A, Lioncino M, Maratea A (2022). Clinical manifestations of 22q11.2 deletion syndrome. Heart Fail Clin.

[4] Morrow BE, McDonald-McGinn DM, Emanuel BS, Vermeesch JR, Scambler PJ (2018). Molecular genetics of 22q11.2 deletion syndrome. Am J Med Genet A.

[5] Solot CB, Knightly C, Handler SD (2000). Communication disorders in the 22Q11.2 microdeletion syndrome. J Commun Disord.

[6] Papamichail M, Pizanias M, Heaton N (2018). Congenital portosystemic venous shunt. Eur J Pediatr.

[7] Guérin F, Blanc T, Gauthier F, Abella SF, Branchereau S (2012). Congenital portosystemic vascular malformations. Semin Pediatr Surg.

[8] Bernard O, Franchi-Abella S, Branchereau S, Pariente D, Gauthier F, Jacquemin E (2012). Congenital portosystemic shunts in children: recognition, evaluation, and management. Semin Liver Dis.

[9] Sokollik C, Bandsma RH, Gana JC, van den Heuvel M, Ling SC (2013). Congenital portosystemic shunt: characterization of a multisystem disease. J Pediatr Gastroenterol Nutr.

[10] Swillen A, Feys H, Adriaens T (2005). Early motor development in young children with 22q.11 deletion syndrome and a conotruncal heart defect. Dev Med Child Neurol.

[11] Schneider M, Debbané M, Bassett AS (2014). Psychiatric disorders from childhood to adulthood in 22q11.2 deletion syndrome: results from the international consortium on brain and behavior in 22q11.2 deletion syndrome. Am J Psychiatry.

[12] Francois B, Lachaux A, Gottrand F, De Smet S (2018). Prenatally diagnosed congenital portosystemic shunts. J Matern Fetal Neonatal Med.

[13] McLin VA, Franchi Abella S, Debray D (2019). Congenital portosystemic shunts: current diagnosis and management. J Pediatr Gastroenterol Nutr.

[14] Lambert V, Ladarre D, Fortas F (2021). Cardiovascular disorders in patients with congenital portosystemic shunts: 23 years of experience in a tertiary referral Centre. Arch Cardiovasc Dis.

[15] Matsumoto T, Okano R, Sakura N (1993). Hypergalactosaemia in a patient with portal-hepatic venous and hepatic arterio-venous shunts detected by neonatal screening. Eur J Pediatr.

[16] Ono H, Mawatari H, Mizoguchi N, Eguchi T, Sakura N (1998). Clinical features and outcome of eight infants with intrahepatic Porto-venous shunts detected in neonatal screening for galactosaemia. Acta Paediatr.

[17] Nishimura Y, Tajima G, Dwi Bahagia A (2004). Differential diagnosis of neonatal mild hypergalactosaemia detected by mass screening: clinical significance of portal vein imaging. J Inherit Metab Dis.

[18] Uchino T, Matsuda I, Endo F (1999). The long-term prognosis of congenital portosystemic venous shunt. J Pediatr.

[19] Stringer MD (2008). The clinical anatomy of congenital portosystemic venous shunts. Clin Anat.

[20] Yoshimoto Y, Shimizu R, Saeki T (2004). Patent ductus venosus in children: a case report and review of the literature. J Pediatr Surg.

[21] Uchino A, Noguchi T, Nomiyama K (2007). Manganese accumulation in the brain: MR imaging. Neuroradiology..

[22] Paganelli M, Lipsich JE, Sciveres M, Alvarez F (2015). Predisposing factors for spontaneous closure of congenital portosystemic shunts. J Pediatr.

[23] Papamichail M, Ali A, Quaglia A, Karani J, Heaton N (2016). Liver resection for the treatment of a congenital intrahepatic portosystemic venous shunt. Hepatobiliary Pancreat Dis Int.

[24] Golewale N, Paltiel HJ, Fishman SJ, Alomari AI (2010). Portal vascular anomalies in Down syndrome: spectrum of clinical presentation and management approach. J Pediatr Surg.

